# Analysis of Telomere Maintenance Related Genes Reveals *NOP10* as a New Metastatic-Risk Marker in Pheochromocytoma/Paraganglioma

**DOI:** 10.3390/cancers13194758

**Published:** 2021-09-23

**Authors:** María Monteagudo, Paula Martínez, Luis J. Leandro-García, Ángel M. Martínez-Montes, Bruna Calsina, Marta Pulgarín-Alfaro, Alberto Díaz-Talavera, Sara Mellid, Rocío Letón, Eduardo Gil, Manuel Pérez-Martínez, Diego Megías, Raúl Torres-Ruiz, Sandra Rodriguez-Perales, Patricia González, Eduardo Caleiras, Scherezade Jiménez-Villa, Giovanna Roncador, Cristina Álvarez-Escolá, Rita M. Regojo, María Calatayud, Sonsoles Guadalix, Maria Currás-Freixes, Elena Rapizzi, Letizia Canu, Svenja Nölting, Hanna Remde, Martin Fassnacht, Nicole Bechmann, Graeme Eisenhofer, Massimo Mannelli, Felix Beuschlein, Marcus Quinkler, Cristina Rodríguez-Antona, Alberto Cascón, María A. Blasco, Cristina Montero-Conde, Mercedes Robledo

**Affiliations:** 1 Hereditary Endocrine Cancer Group, Human Cancer Genetics Program, Spanish National Cancer Research Centre (CNIO), 28029 Madrid, Spain; mmonteagudo@cnio.es (M.M.); ljleandro@cnio.es (L.J.L.-G.); ammontes@cnio.es (Á.M.M.-M.); bcalsina@cnio.es (B.C.); mpulgarin@cnic.es (M.P.-A.); adiazt@ext.cnio.es (A.D.-T.); smellid@cnio.es (S.M.); rleton@cnio.es (R.L.); edgilv@cnio.es (E.G.); crodriguez@cnio.es (C.R.-A.); acascon@cnio.es (A.C.); cmontero@cnio.es (C.M.-C.); 2 PhD Program in Neuroscience, Autonoma de Madrid University, 28029 Madrid, Spain; 3 Telomeres and telomerase Group, Molecular Oncology Program, Spanish National Cancer Research Centre (CNIO), 28029 Madrid, Spain; pmartinez@cnio.es (P.M.); mblasco@cnio.es (M.A.B.); 4 Centro de Investigación Biomédica en Red de Enfermedades Raras (CIBERER), 28029 Madrid, Spain; 5 Confocal Microscopy Core Unit, Biotechnology Program, Spanish National Cancer Research Centre (CNIO), 28029 Madrid, Spain; mperez@cnio.es (M.P.-M.); dmegias@cnio.es (D.M.); 6 Molecular Citogenetic, Unit Human Cancer Genetics Program, Spanish National Cancer Research Centre (CNIO), 28029 Madrid, Spain; rtorresr@cnio.es (R.T.-R.); srodriguezp@cnio.es (S.R.-P.); 7 Histopathology Core Unit, Biotechnology Program, Spanish National Cancer Research Centre (CNIO), 28029 Madrid, Spain; pgonzalez@cnio.es (P.G.); ejcaleiras@cnio.es (E.C.); 8 Monoclonal Antibodies Core Unit, Biotechnology Program, Spanish National Cancer Research Centre (CNIO), 28029 Madrid, Spain; sjimenez@cnio.es (S.J.-V.); groncador@cnio.es (G.R.); 9 Department of Endocrinology, La Paz University Hospital, 28046 Madrid, Spain; calvareze@salud.madrid.org; 10 Department of Pathology La Paz University Hospital, 28046 Madrid, Spain; ritamaria.regojo@salud.madrid.org; 11 Department of Endocrinology, 12 de Octubre University Hospital, 28041 Madrid, Spain; maria.calatayud@salud.madrid.org (M.C.); sonsoguadalix@gmail.com (S.G.); 12 Department of Endocrinology, Clínica Universidad de Navarra, 28027 Madrid, Spain; mcurras@unav.es; 13 Department of Experimental and Clinical Medicine, University of Florence, 50121 Florence, Italy; elena.rapizzi@unifi.it (E.R.); letizia.canu@unifi.it (L.C.); massimo.mannelli@unifi.it (M.M.); 14 Medizinische Klinik und Poliklinik IV, Klinikum der Universität München, 80336 Munich, Germany; Svenja.Noelting@usz.ch (S.N.); Felix.Beuschlein@usz.ch (F.B.); 15 Department of Internal Medicine I, Division of Endocrinology and Diabetes, University Hospital Würzburg, University of Würzburg, 97070 Würzburg, Germany; remde_h@ukw.de (H.R.); fassnacht_m@ukw.de (M.F.); 16 Comprehensive Cancer Center, Mainfranken University of Würzburg, 97070 Würzburg, Germany; 17 Institute of Clinical Chemistry and Laboratory Medicine, University Hospital Carl Gustav Carus, Technische Universität Dresden, 01069 Dresden, Germany; nicole.bechmann@uniklinikum-dresden.de (N.B.); graeme.eisenhofer@uniklinikum-dresden.de (G.E.); 18 Department of Medicine III, University Hospital Carl Gustav Carus, Technische Universität Dresden, 01069 Dresden, Germany; 19 Klinik für Endokrinologie Diabetologie und Klinische Ernährung, Universitätsspital Zürich, 8091 Zürich, Switzerland; 20 Endocrinology in Charlottenburg Stuttgarter Platz 1, 10627 Berlin, Germany; marcusquinkler@t-online.de

**Keywords:** pheochromocytoma, paraganglioma, PPGL, telomeres, *TERT*, *ATRX*, *NOP10*, prognostic biomarker, ALT

## Abstract

**Simple Summary:**

Telomere maintenance involving *TERT* and *ATRX* genes has been recently described in metastatic pheochromocytoma and paraganglioma, reinforcing the importance of immortalization mechanisms in the progression of these tumors. Thus, the aim of this study was to analyze additional telomere-related genes to uncover potential new markers capable of identifying metastatic-risk patients more accurately. After analyzing 29 telomere-related genes, we were able to validate the predictive value of *TERT* and *ATRX* in mPPGL progression. In addition, we were able to identify *NOP10* as a novel prognostic risk marker of mPPGLs, which also facilitates telomerase-dependent telomere length maintenance in these tumors. Interestingly, NOP10 overexpression assessment by IHC could be easily included within the current battery of markers for stratifying PPGL patients to fine-tune their clinical diagnoses.

**Abstract:**

One of the main problems we face with PPGL is the lack of molecular markers capable of predicting the development of metastases in patients. Telomere-related genes, such as *TERT* and *ATRX,* have been recently described in PPGL, supporting the association between the activation of immortalization mechanisms and disease progression. However, the contribution of other genes involving telomere preservation machinery has not been previously investigated. In this work, we aimed to analyze the prognostic value of a comprehensive set of genes involved in telomere maintenance. For this study, we collected 165 PPGL samples (97 non-metastatic/63 metastatic), genetically characterized, in which the expression of 29 genes of interest was studied by NGS. Three of the 29 genes studied, *TERT*, *ATRX* and *NOP10*, showed differential expression between metastatic and non-metastatic cases, and alterations in these genes were associated with a shorter time to progression, independent of *SDHB*-status. We studied telomere length by Q-FISH in patient samples and in an in vitro model. *NOP10* overexpressing tumors displayed an intermediate-length telomere phenotype without ALT, and in vitro results suggest that *NOP10* has a role in telomerase-dependent telomere maintenance. We also propose the implementation of NOP10 IHC to better stratify PPGL patients.

## 1. Introduction

Pheochromocytomas (PCC) and paragangliomas (PGL), all together called PPGLs, are rare neuroendocrine tumors derived from the adrenal medulla or extra-adrenal paraganglia [1]. PPGLs are known as the most hereditary neoplasms, since at least 40% are caused by germline mutations in one of the 23 genes associated so far with the susceptibility to develop this kind of tumor [2]. In addition, 30–40% of PPGLs are due to somatic mutations in these same genes, other cancer-related genes or chromosomal translocations involving the *MAML3* gene [3].

Approximately 15–20% of the patients develop metastatic disease (mPPGL) in the first two-three years after diagnosis [4,5]. In this regard, it is important to note that although synchronous metastases occur in 35–50% of cases, metachronous lesions can be developed during the decade following the initial diagnosis [4]. Prognosis of mPPGL is poor and heterogeneous, showing a 5-year overall survival of 40–77% from diagnosis of the first metastasis [6].

Risk factors associated with metastatic disease in PPGLs are scarce, inaccurate and remain poorly defined, mainly due to the low prevalence of the disease, which makes it difficult to recruit large series of patients to reach robust conclusions. Therefore, the early detection of mPPGLs becomes highly relevant for early detection of metastatic disease for which treatment options and therapies remain limited for these patients beyond surgery [7,8,9,10,11].

Even so, there are some clinical features that provide useful information about the potential for developing metastases, such as transcriptional clusters, tumor size and location or plasma metabolites concentration [3,7,8,9,10,11]. Among molecular metastatic risk markers, it is accepted that *SDHB* mutations are associated with poor prognosis [12]. Although, it has been suggested that additional factors must be involved in disease progression [13]. Recent studies reported that immortalization mechanisms common in other types of carcinomas, which involve telomere deregulation, also play a role in PPGL progression. In fact, the activation of the telomerase gene, *TERT*, and *ATRX* loss of function mutations have been reported to be associated with poor prognosis in PPGL [3,14,15].

Telomeres are DNA regions associated with the shelterin protein complex located at the end of chromosomes. The function of these structures is to protect the DNA *termini* from degradation and from being recognized as DNA double-strand breaks (DSB), to prevent end-to-end interchromosomal fusions [16,17,18,19,20]. Telomeric regions shorten with each cell division [21,22], due to the “end replication” problem and other processes, such as DNA processing and oxidative damage [16,17]. When they reach a critical short length, cells become senescent/quiescent, affecting the generative capacity of tissues [23]. Telomere shortening can be compensated through the *de novo* addition of telomeric repeats by telomerase, a reverse transcriptase composed of a catalytic subunit (TERT) and an RNA component (TERC), used as a template for telomere elongation [24]. *TERT* is downregulated in the majority of tissues post-natally, with the exception of adult stem cells [25]. Noteworthy, human tumors reach an indefinite proliferative capacity by either upregulating telomerase or activating the alternative lengthening of the telomeres mechanism [15,26,27,28].

The enzyme telomerase (TERT/TERC) is associated with additional factors that are required for telomerase biogenesis, localization and activity in vivo. Among other factors, telomerase forms a complex with the H/ACA-motif RNA-binding proteins, i.e., DKC1, NOP10, GAR1 and NHP2, that are involved in the proper stability, regulation and intracellular trafficking of telomerase and therefore are key for telomerase-dependent telomere lengthening [29,30].

Since telomere regulation is an important event in the metastatic progression of PPGLs, the aim of this study was to analyze other genes directly or indirectly related to telomere maintenance, in order to uncover potential new markers capable of identifying PPGL patients at risk of developing metastatic disease more accurately. For this purpose, we performed an exhaustive analysis of the expression of 29 genes related to telomere maintenance in a series of 165 metastatic and non-metastatic PPGL tumor samples with clinical and genetic information. The 29 telomere-related genes, henceforth called *telomerome*, are grouped into different categories: telomerase holoenzyme complex, shelterin complex, ALT (alternative lengthening of telomeres) phenotype and genes indirectly related to telomere maintenance. We were able to validate the predictive value of *TERT* and *ATRX* for mPPGL. Furthermore, our findings from patient samples showed that *NOP10* is a novel prognostic risk marker of developing mPPGLs. On the other hand, *in vitro* experiments supported a mechanism in which *NOP10* overexpression facilitates telomerase-dependent telomere length maintenance in these tumors.

## 2. Materials and Methods

### 2.1. PPGL Cohort and Genetic Characterization

The CNIO study cohort included 149 patients: 81 women, 64 men and 4 of unknown gender, with a mean age at diagnosis of 45 years and a mean follow-up time of 6 years. Among patients, 47 were classified as metastatic (from which 54 primary tumors and 9 metastases were available), diagnosed either with synchronous or metachronous metastasis. This series also included 5 patients with tumors classified as clinically aggressive, characterized by detection of capsular, vascular or adipose tissue infiltration in the pathology report, and/or multiple local recurrences, but without confirmed metastases. The 97 remaining tumors corresponded to non-metastatic PPGL patients with a mean follow-up of 7.67 years (2800 days; min: 0-max: 5895). The series included 96 formalin-fixed paraffin embedded tissues (FFPE) and 69 frozen samples. Clinical data are summarized in Table 1.

**Figure 1:**
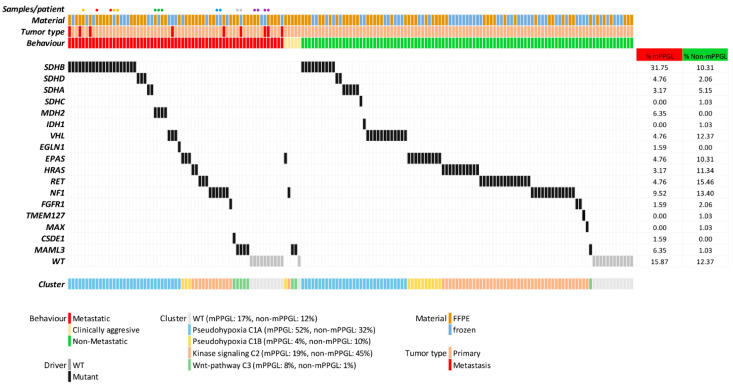
CNIO discovery series: genetic characterization of PPGL series: tumor driver gene and cluster classification. The frequency of each driver gene (percentage) per group, material for each sample and tumor tissue type (primary/metastasis) are shown in the figure. The colored dots represent tumors from the same patient; each color corresponds to a different patient.

Germline and somatic mutational status characterization were performed using a customized NGS panel. The targeted gene panel was designed using the AmpliSeq Custom DNA Panel (Illumina, San Diego, CA, USA), and included the main susceptibility PPGL genes (*VHL, RET, SDHA, SDHB, SDHC, SDHD, SDHAF2, SDHAF1, MAX, HIF1A (*exon *12), HIF2A (exon 12), TMEM127, HRAS, KRAS, NF1, GOT2, FH, MDH2, SLC25A11, DNMT3A (*exon *8), DLST (*exon *14), MERTK (*exon *17), IDH1, IDH2, CSDE1, EGLN1, EGLN2, BRAF (*exon *15), MET (*exons *14*–*21), FGFR1 (*exons *12* and *14), KIF1B, CDKN1B, MEN1, PTEN, H3F3a*) and *ATRX*. The panel was used according to the manufacturer’s instructions, starting with 200ng of DNA. Interpretation of variants was performed following the recommendations of the NGS in PPGL Study Group and the American College of Medical Genetics and Genomics-Association for Molecular Pathology (ACMG-AMP) [31,32], and mutations detected were confirmed by Sanger sequencing (Figure 1).

Written informed consent for the use of specimens and clinical data were obtained from all patients, according to the institutional ethics committee guidelines. All subjects gave their informed consent for inclusion before they participated in the study. The study was conducted in accordance with the Declaration of Helsinki, and the protocol was approved by the following ethics committees: Hospital Universitario 12 de Octubre (15/024), Madrid, Spain; Universität Spital (2017-00771), Zurich, Germany; Klinikum der Universität (379-10), Munich, Germany; University Hospital Würzburg (ENS@T Ethics Committee 88/11), Würzburg, Germany; Azienda Ospedaliera Universitaria Careggi (Prot. N. 2011/0020149) Florence, Italy; Berlin Chamber of Physicians (Eth-S-R/14), Berlin, Germany.

### 2.2. Tumor DNA Extraction

Total DNA was isolated from FFPE samples using the Maxwell^®^ RSC DNA Formalin-fixed paraffin embedded Kit (Promega, Madison, WI, USA) and a Maxwell^®^ RSC Instrument (Promega). DNA from frozen tissue was extracted with DNeasy^®^ Blood and Tissue Kit (Qiagen, Hilden, Germany), following the manufacturer’s protocols. In FFPE, at least 2 cores were obtained from selected tumor areas. DNA was quantified using QuantiFluor^®^ ONE dsDNA System kit (Promega) or Quant-iT^TM^ PicoGreen^TM^ dsDNA protocol (Invitrogen, Carlsbad, CA, USA).

### 2.3. Tumor RNA Extraction and Quality Test

Three or four 5 μm sections, or at least 2 cores from tumor enriched areas, were used for total RNA extraction from FFPE specimens using Maxwell^®^ RSC RNA FFPE Kit (Promega). Frozen sample RNA extraction was performed using TRIzol^TM^ reagent (Invitrogen) following manufacturers’ protocol. After extraction, RNA was quantified by Nanodrop (NanoDrop™, Waltham, MA, USA). RNA integrity was assessed using Agilent Bioanalyzer 2100 (Agilent Technologies, Santa Clara, CA, USA) and the percentage of RNA fragments over 200 nt (DV200) was determined. RNA input was adjusted to DV_200_ values according to the following criteria: DV_200_ > 70%, 200 ng; DV_200_ = 50–70%, 400 ng; DV_200_ = 30–50%, 600 ng, and poor integrity RNAs (DV_200_ < 30%) were discarded. High quality commercial RNA from human placenta tissue and RNAs from human cancer cell lines were included in all runs as inter-assay controls. Three frozen/FFPE pairs of tumors, for which both types of preservation were available, were included to evaluate technique reliability for samples with different RNA integrity.

### 2.4. TREx RNA Sequencing

A customized TruSeq Targeted RNA expression (TREx) panel (Illumina, San Diego, CA, USA), capable of analyzing paraffin-embedded and frozen tissues, was designed to assess telomere-related gene expressions. A total of 29 telomere maintenance genes were included in the assay, belonging directly and indirectly to telomere maintenance pathways: telomerase complex (*TERT*, *TERC*, *DKC1*, *GAR1*, *NOP10*, *NHP2*); shelterin–telosome protein complex (*POT1*, *TERF1*, *TERF2*, *TERF2IP*, *TINF2*, *TPP1*); histone binding and alternative telomere lengthening mechanism (*ATRX*, *DAXX*, *TNKS*); *non*-canonical telomere maintenance (*ACD*, *FBXO4*, *GPX2*, *MCRS1*, *MKRN1*, *NAT10*, *NFX1*, *RLIM*, *SMG5*, *SMG6*, *SOX7*, *TEP1*, *WRAP53*, *YLPM1*).

Sequencing runs of 150bp single-end reads were successfully performed in an Illumina MiSeq system. The output data were mapped to the reference genome version GRCh37, adapted for the TREx custom panel, using TopHat [33] included in the Nextpresso suite [34]. Random down-sampling of each sample was performed to obtain a final number of ≈170,000 mapped reads. Samples with less than 170,000 aligned reads were discarded due to low read depth (<700 reads/amplicon). A total of 165 samples (96 FFPE and 69 frozen) passed this cut-off and were used for the analysis: 143 samples had >1000 reads/amplicon, 23 samples had between 1000–750 reads/amplicon and 4 were excluded with <750 reads/amplicon. New mapping process and different quality steps were performed with the down-sampled FASTQ files. Briefly, to decrease the bias effect between FFPE and frozen samples, we used limma package [35], which allowed obtaining unbiased log2CPMs (counts per million) for all the selected samples.

*TERT* expression was detected using 3 specific probes, and only samples with at least 3 raw counts in each one of the probes and average raw counts ≥ 4 were considered as positive for expression, in order to minimize false positive identification due to *TERT* low-expressor condition.

### 2.5. ATRX Mutations

Mutations in *ATRX* were detected by the customized NGS panel in a set of 120 tumors. Exome data were available for 45 additional PPGLs (unpublished data). WES (whole exome sequencing) was performed using two different Illumina sequencing platforms, HiSeq and NovaSeq, generating 100bp paired-end reads. RubioSeq suite was used for the exome analysis [36], and data were processed and aligned to the human reference genome GRCh37 using Burrows–Wheeler Aligner (BWA). Germline and somatic variants were detected with Haplotype Caller [37] and MuTect [38] (Table S1).

### 2.6. Identification of TERT PROMOTER Mutations (TPMs)

Mutations causing altered *TERT* expression were studied using NGS, following the 16S Metagenomic Sequencing Library Preparation (Illumina, San Diego, CA, USA) protocol. To identify TPMs (chr5:1,295,228 C > T and chr5: 1,295,250 C > T), amplicons of 151bp were amplified from 50 ng of tumor DNA (primer sequences provided in Table S2). Amplicon PCR was performed using Multiplex QIAGEN 2X Master Mix following manufacturer’s instructions. Index PCR was later executed with the EasyTaq DNA polymerase (TransGen Biotech, Beijing, China) using synthetic indices from the Nextera XT Index Kit (Illumina, San Diego, CA, USA). Both PCR products were purified using AMPure XP beads (Beckman Coulter, Pasadena, IN, USA) and quantified by PicoGreen (Thermo Fisher Scientific, Waltham, MA, USA). Amplicon concentration was normalized and pooled up to 96 samples for a single run.

Libraries were sequenced according to manufacturer’s instructions in a MiSeq sequencer (Illumina). The sequencing module used was the “PCR Amplicon” protocol with a paired-end design with 150 base pairs reads. Illumina software was used to perform the variant calling, and Illumina VariantStudio software (Illumina) was used to obtain the sequencing results. *TERT* promoter mutations detected by NGS were confirmed by PCR and Sanger sequencing (primers are provided in Table S2).

### 2.7. Analysis of TERT Promoter Methylation Levels

To analyze THOR (*TERT* hypermethylated oncological region) methylation levels, bisulfite-modified DNA was used to amplify four THOR sections: A1, A2, A3 and A4, as described in Lee et al., 2019. Within the fourth THOR amplicon is located the UTSS region (upstream of the transcription start site), which contains a subset of five CpG sites (CpG 1295586, 1295590, 1295593, 1295605 & 1295618) whose average methylation level accurately correlates with the average methylation level of the whole THOR.

An amount of 100 ng of tumor DNA was bisulfite-modified using the EZ-96 DNA MethylationTM Kit (ZYMO RESEARCH, Irvine, CA, USA) research for the analysis of promoter hypermethylation. Bisulfite-modified DNA results in the conversion of unmethylated cytosine to uracil, which will be copied as thymine upon PCR, thus distinguishing methylated (thymine) from unmethylated (cytosine) DNA bases. Preparation and sequencing of the four THOR PCR amplicons were performed following the protocol “16S Metagenomic Sequencing Library Preparation” for the Illumina MiSeq platform with a paired-end design of 150 base pairs reads. Primers were chosen according to Lee et al., 2019 (Table S2).

Paired-end FASTQ files of each sample were generated. Only forward reads were used in the analysis. Trimming was performed with cut-adapt software to eliminate the sequences corresponding to the Illumina adapters incorporated during the sequencing process. The first step was to generate a reference genome adapted to bisulfite modification from the human genome assembly hg19. Reads were aligned to this modified reference genome using BS-Seeker2 software (2), taking into account changes introduced by bisulfite modification and favoring correct alignment. Secondly, we obtained the coverage at the positions of interest (UTSS region) using bam-readcount software (https://github.com/genome/bam-readcount, accessed on 16 January 2019). Finally, we calculated for each CpG site of interest the percentage of methylation observed as a function of the number of reads showing “C” or “T” at that position. Only samples with a mean UTSS-region value ≥ 16.1% were considered as hypermethylated, as previously established by Lee et al., 2019.

### 2.8. TERT Copy Number Alterations (CNAs)

*TERT* CNAs analysis was performed in those samples from which WES data were available (unpublished data). Anaconda pipeline was used to detect somatic copy number alterations [39]. CNA profiles at gene level were identified using GISTIC 2.tool [40]. Data from frozen and FFPE samples were analyzed separately to minimize the preservation type bias in the analysis. Thresholds for gain detection were set to 4 and 8 for frozen and FFPE samples, respectively.

### 2.9. Telomerome Significant Genes and Metastasis Prediction Risk Model

To determine tumors with altered expression of telomere maintenance genes, we estimated the interquartile range (IQR) of the expression values of the non-metastatic samples with long follow-up (≥8 years) from diagnosis (*n* = 45). We considered de-regulated gene expression tumors those with values below or above the threshold set using lower/upper whiskers (Q1 − (1.5 × IQR) or Q3 + (1.5 × IQR), respectively) of the gene expression dispersion. Candidate genes were chosen according to Fisher exact test after Bonferroni correction. Expression outlier data of candidate genes were transformed into dichotomous variables. For this analysis, tumors with clinically aggressive phenotype (*n* = 5) and non-metastatic samples with <8 years or unknown follow-up (*n* = 52) were excluded, leaving a total of 108 samples (63 metastatic and 45 non-metastatic).

A logistic regression analysis to assess the odds of metastatic risk was executed including as variables *SDHB*, *TERT*/*ATRX*, *NOP10* and *FBXO4.* Selection of the best gene classifier was evaluated using a stepwise conditional logistic regression model. Non-metastatic patients with unknown follow-up, and those with clinically aggressive tumors, were excluded from the analysis.

The classification power of *telomerome* genes selected in the previous step was evaluated by computing receiver operating characteristic (ROC) curves and area under the curve analysis (AUC). A total number of 45 non-metastatic (≥8 years of follow-up) and 54 primary-metastatic samples were included. This analysis was applied considering 3 scenarios: 1) tumors with any event in *TERT* (expression outliers, TPMs, promoter hypermethylation or gains in 5p region) and/or *ATRX* (expression outliers and loss of function mutations), 2) tumors with only outlier expression of *NOP10* but excluding events in *TERT* and *ATRX*, and 3) considering any event in any of the 3 aforementioned *telomerome* genes. Data were analyzed using IBM-SPSS Version 19 (Armonk, NY, USA) and GraphPad Prism Version 5 (San Diego, CA, USA).

### 2.10. Time to Progression and Validated Telomerome Genes

Time to progression was evaluated using the Kaplan–Meier analysis for the whole series with follow-up data, testing differences using the log-rank test (IBM-SPSS Version 19) Metastasis (*n* = 9), clinically aggressive (*n* = 5) and non-metastatic cases with unknown follow-up (*n* = 6) were excluded from the analysis. *TERT*+*ATRX* (considering *TERT* expression outliers, TPMs, *TERT* promoter hypermethylation, gains and *ATRX* down expression outliers and loss of function mutations) and *TERT*+*ATRX*+*NOP10* (considering *NOP10* overexpression outliers) were studied for association with time to progression. The latter was defined as the number of days between surgery of the primary PPGL and the appearance of the first confirmed metastasis. The inclusion criteria were the presence of either synchronous or metachronous metastases (those that appeared before and after one year since surgery of the primary tumor, respectively) or at least 2 years’ follow-up in the case of non-metastatic patients. Patients with non-metastatic tumors were censored at the date of last follow-up available.

### 2.11. Telomere Length Q-FISH, High-Throughput Quantification

Telomere length was studied by Q-FISH in selected representative FFPE samples. Hematoxylin and eosin-stained tumor sections were evaluated by a pathologist in order to select the areas of interest. Samples with a high tumor content were cut into complete sections (4 µm), and for those samples with a low tumor content, representative cores (1 mm) were selected for study in a tissue micro array (TMA). After deparaffinization and rehydration, tissues were washed in PBS 1X and fixed in 4% formaldehyde for 5 min. After washing, slides were dehydrated in a 70–90%–100% ethanol series (5 min each).

Slides were air dried and 30 µL of the telomere probe mix (10 mM TrisHCl, pH 7.2, 25 mM MgCl_2_, 9 mM citric acid, 82 mM Na_2_HPO_4_, 50% deionized formamide (Sigma-Aldrich, Darmstadt, Germany), 0.25% blocking reagent (Roche, Basel, Switzerland), and 0.5 µg/mL Telomeric PNA probe (Panagene, Daejeon, Korea) was added. Slides were incubated for 3 min at 85 °C and then 2 h at room temperature in a wet chamber in the dark. Slides were washed twice for 15 min each in 10 mM TrisCl (pH 7.2) and 0.1% BSA in 50% formamide and then three times for 5 min each in TBS 0.08% Tween 20. After washing, slides were stained with DAPI (0.2 µg/mL) and dehydrated in a 70–90%–100% ethanol series. Dried samples were finally mounted with VECTASHIELD mounting media (Vector Laboratories, Burlingame, CA, USA).

Telomere length analysis is based on the specific and stable hybridization of the PNA with the telomeric region; the intensity of this PNA is directly related to telomere length allowing the measurement of telomeres at each individual chromosome end. Samples were imaged and quantified by confocal microscopy. For each sample evaluation, five representative areas from each tumor were imaged for an unbiased study of telomere length. Q-FISH images were acquired in a confocal microscope equipped with a 63×/NA 1.4 oil immersion objective and LAS AF v2.6 software (Leica-Microsystems, Wetzlar, Germany), and maximum projection images were created with the LAS AF 2.7.3.9723 software. Telomere signal intensity from Z-stacks was quantified using Definiens Developer Cell software version XD 64 2.5. Telomere length was estimated as the mean telomere intensity value per nucleus.

### 2.12. Promelocytic Leukaemia (PML) Bodies and Telomere Co-Localization by Immuno-Q-FISH

FFPE tissue samples were fixed in 10% buffered formalin, dehydrated, embedded in paraffin wax and sectioned at 4 μm. Tissue sections were deparaffinized in xylene and re-hydrated through a series of decreasing ethanol concentrations up to water. Immunofluorescence (IF) was performed on deparaffined tissue sections processed with 10 mM sodium citrate (pH 6.5) cooked under pressure for 2 min. Tissue sections were permeabilized with 0.5% Triton in PBS and blocked with 5% BSA in PBS. Samples were incubated overnight at 4 ºC with rabbit polyclonal anti-PML (1:100; Santa Cruz Biotechnology, Santa Cruz, CA, USA, H-238). Q-FISH was performed on IF-stained slides fixed with 4% formaldehyde for 20 min. The DAPI images were used to detect telomeric signals inside each nucleus. Immunofluorescence images were obtained with a TCS-SP8 STED 3X confocal microscope equipped with a 63×/NA 1.4 oil immersion objective, a white light laser and LAS X v3.5 software (Leica-Microsystems). Z-stacks of the samples were acquired and then analyzed with Definiens Developer XD 64 v2.5 software (Definiens Inc., Munich, Bayern, Germany).

### 2.13. Characterization of NOP10 Expression Mechanisms

The coding regions (exons 1 and 2) of *NOP10* gene were analyzed by Sanger sequencing in order to detect activating mutations (primers in Table S2). Additionally, *NOP10* promoter region (200 bp upstream of the transcription start site or TSS) was checked for activating mutations using the previously mentioned WES data. Epigenetic mechanisms were also studied, including methylation 450K array data from PPGL TCGA project ([3], https://xenabrowser.net/datapages/, accessed on 19 February 2021) and PPGL CNIO series previously published data [41,42,43,44] as well as miRNA expression data from PPGL TCGA project (https://xenabrowser.net/datapages/, accessed on 19 February 2021) and from the PPGL CNIO series [45].

NOP10 protein expression was assessed by immunohistochemistry (IHC) in metastatic and non-metastatic PPGLs, previously selected according to *NOP10* overexpression. Sections of 2 μm thick were prepared from FFPE tissue and were dried in a 60 °C oven overnight. The sections were placed in a BOND-MAX Automated Immunohistochemistry Vision Biosystem (Leica Microsystems GmbH, Wetzlar, Germany) using standard protocol. First, tissues were deparaffinized and pre-treated with the Epitope Retrieval Solution 2 (EDTA-buffer pH8.8) at 98 °C for 20 min. After washing steps, peroxidase blocking was carried out for 10 min using the Bond Polymer Refine Detection Kit DC9800 (Leica Microsystems GmbH). Tissues were again washed and then incubated with the primary antibody anti-NOP10 (rabbit monoclonal antibody (EPR8857) (ab134902, Abcam)) diluted 1:1000 for 30 min. Subsequently, tissues were incubated with polymer for 10 min and developed with DAB-chromogen for 10 min. Human kidney slides were used as positive staining control following manufacturer’s recommendations. Additionally, patients with long-term follow-up (>8 years), *TERT* over-expressing samples and *ATRX* down-expressing samples and normal adrenal medulla FFPE slides were included as controls.

Images of whole sections were taken with a slide scanner (AxioScan Z1, Zeiss, Jena, Germany). For analysis, an appropriate script was created using QuPath software (Belfast, UK) [46]. Representative areas from each slide were chosen for quantification program training, creating an appropriate script for *NOP10* antibody according to the intensity method: positivity was evaluated in three stages from high to low (3+, 2+, 1+) and negative. After training and script optimization, the quantification step was run, and results were exported as excel files with scoring data for each file.

Staining was classified as: low staining (negative and 1+) and high staining (2+ and 3+). Tumor staining was compared with negative staining from normal adrenal medulla. The percentage of high positivity staining was compared between samples (Neuwman–Keuls multiple comparison test, *p*-value < 0.05).

### 2.14. Cell Culture and Generation of TERT and NOP10 Overexpression Models

Human mesenchymal cells [47] were cultured in MesenPRO RS™ (Gibco) medium with L-Glutamin (5%; Gibco) and penicillin/streptomycin (1%; Gibco). Cells were maintained in monolayer in an incubator at 37 °C and 5% CO_2_. For the experiments performed *in vitro*, two different plasmids were used:

1. *pLV-TERT-IRES-puro*: to generate *TERT* overexpressing cells, lentiviral plasmid pLV-TERT-IRES-hygro was acquired from Addgene repository (Addgene Plasmid #85140 [48]). Selection antibiotic was changed from hygromycin to puromycin.
2. *pLV-NOP10-IRES-hygro*: *NOP10* expression plasmid (NM_018648) was acquired from OriGene (CAT#: RC209038). Using pLV-TERT-IRES-hygro backbone, *TERT* gene was replaced by *NOP10* ORF sequence from the aforementioned plasmid, generating a new pLV-*NOP10*-IRES-hygro vector.

Lentiviral plasmids were introduced in HEK293T cells (CRL-1573, ATCC) [47] using lipofectamine (Thermo Fisher). After cell culture, supernatant-carrying lentiviral particles were collected and used for mesenchymal cell infection. The infection was performed by using a small volume from the viral supernatant, allowing viral particles to physically contact mesenchymal cells. Once cells were selected with their respective antibiotics (Puromycin 0.35 µg/mL, Gibco; Hygromicin: 20 µg/mL; Invitrogen), overexpression of both *TERT* and *NOP10* was confirmed by RT-PCR. Briefly, each cell line was seeded in 60 mm plates. After expansion (3 × 10^6^ cells), RNA was isolated using TRIzol Reagent^®^ (Ambion-Life Technologies, Waltham, MA, USA) following the manufacturer’s instructions. cDNAs were prepared from 500 ng of RNA using the qScriptTM cDNA Synthesis Kit (Quanta Biosciences, Gaithersburg, MD, USA) and mRNA levels were quantified by real-time PCR using the Universal ProbeLibrary set (Roche), as described by the vendor, on a QuantStudio 6 Flex Real-Time PCR System (Applied Biosystems, Waltham, MA, USA) using TaqMan^®^ Universal PCR Master Mix No AmpErase^®^ UNG (Applied Biosystems). Normalization was carried out with the *β-ACTIN* housekeeping gene and relative mRNA levels were estimated by the ΔΔCt method [49]. Primers and probes used for RT-PCR shown in Table S2.

### 2.15. Telomere Length Q-FISH on Cell Spreads

For telomere length analysis, non-confluent hMSC were harvested. Cells were collected by centrifugation and after hypotonic swelling in 0.03 M sodium citrate for 30 min at 37 °C, hMSC were fixed in methanol–acetic acid (3:1). Cell suspension was dropped onto wet microscope slides and dried overnight. After drying, we proceeded to carry out quantitative telomere fluorescence in situ hybridization (Q-FISH), as previously described [50].

## 3. Results

### 3.1. Description of the PPGL-Telomerome Series

The series comprises a collection of 165 tumors, representative of the genetic landscape of the different susceptibility genes in PPGLs. After genetic characterization, 38.78% of tumors (64/165) belonged to cluster C1A, 8.48% (14/165) to cluster C1B, 33.93% (56/165) to C2 and 4.84% (8/165) to C3. Among the 63 mPPGLs, 52.38% (33/63) belong to the C1A cluster, associated with a high risk of progression. The other ones belong to clusters C1B (3/63), C2 (12/63) and C3 (5/63). This series also included 23 WT samples (23/165) (Figure 1, Table 1).

### 3.2. Study of the Telomerome Expression Profile and Outlier Selection

After assessing the telomerome expression data, interquartile range analysis was applied for detecting expression outliers of candidate genes, and the number of outliers was compared between metastatic and long follow-up non-metastatic samples (more than 8 years). A total of 3 out of 29 genes, *TERT*, *NOP10* and *FBXO4*, showed differences between metastatic and non-metastatic PPGLs by Fisher´s exact test after Bonferroni correction. Although not significant, we added *ATRX* as a prognostic marker because it had already been associated with mPPGLs [14], selecting a final number of four candidate genes for further analyses (Figure S1).

### 3.3. Mechanisms That Trigger Aberrant Expression of Telomerome Genes in mPPGL

**Figure 2:**
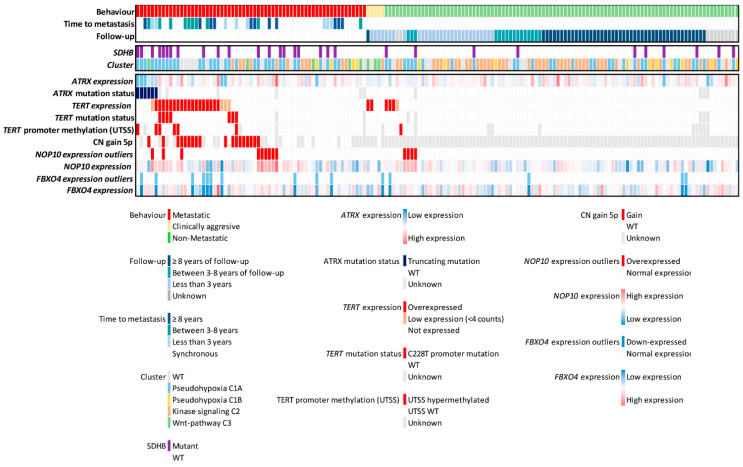
Summary of genomic alterations in PPGL series linked to *telomerome* events. Tumor behavior and patient follow-up are shown, non-metastatic patients mean follow-up = 7.67 years (min: 9 days, max: 36 years). Patients classification was made according to driver mutations. Events in *ATRX* include *ATRX* low expression and *ATRX* loss of function mutations. *TERT* events include: *TERT* overexpression, *TERT* promoter mutation, *TERT* promoter hypermethylation (UTSS median value > 16.1%) and CN gain 5p. *NOP10* and *FBXO4* expression outliers and continuous expression data are shown.

Six tumors carried loss of function mutations in *ATRX* that correlated with a decreased *ATRX* expression, three of them being outliers (Table S1). Five of them corresponded to the pseudohypoxia cluster and the remaining one to the Wnt-pathway cluster (Figure 2).

*TERT* reactivated expression was detected in 23/63 (36.5%) mPPGLs, as well as in two clinically aggressive tumor samples and four non-mPPGLs with short/unknown follow-up (Figure 2). Aberrantly high *TERT* expression could arise through four major mechanisms: enhancing promoter mutations, promoter hypermethylation in the THOR-UTSS (*TERT* hypermethylated oncological region untranscribed site), *TERT* locus amplification and rearrangements involving the super enhancer region located upstream *TERT* TSS and up to 5.4 Mb [51].

The sequencing of the *TERT* promoter from 158 PPGLs with available material revealed seven mPPGLs carrying the C228T mutation, from which five showed reactivation of *TERT* expression. These five mutants with *TERT* overexpression were also carrying *SDHB* driver mutations (5/7, 71.4%) (Figure 2).

*TERT* promoter methylation analysis was performed in 147 tumors with good quality DNA available (Figure S2A). The median hypermethylation value was significantly higher in metastatic samples when compared with non-mPPGLs (mPPGL median 8.34%, SD: 12.7; non-mPPGL median: 3.57%, SD: 3.4; unpaired *t*-test) (Figure S2B). Seven tumors were considered hypermethylated as they showed median UTSS-THOR methylation value over 16.1%, as previously established [51] (Figure S2A,B). Among them, six were mPPGLs, and the remaining case corresponded to a non-metastatic PPGL without follow-up data. Notably, 6/7 (86%) *TERT* promoter hypermethylated cases belong to the C1A cluster. Simultaneous events in the *TERT* promoter (mutation and hypermethylation) were present in one sample (Figure 2).

Among the 44 samples with the copy number data available, we found that gains in the *TERT* locus (5p15.33) were present in 8 out of 18 *TERT* expressing samples (40%) and 10/26 non-expressing samples (38%). *TERT* locus gain overlapped with promoter mutation and/or methylation in 2/18 (11.1%) specimens. Among metastatic samples with *TERT* locus gains, two of them were *SDHB*-mutated (2/18, 11.1%), and 5/18 (27.7%) belonged to the C1A cluster. Among the 50 PPGLs showing any type of *TERT* event, 25 (50%) belonged to the C1A cluster, 1 (0.2%) to cluster C1B, 7 (14%) to C2, 3 (6%) to C3 and 14 (28%) were classified as wild type samples with an unknown driver mutation (Figure 2). Finally, we analyzed the association between events in the *TERT* locus and its expression. CNAs were the event associated with the highest median *TERT* expression (Figure S2C).

**Figure 3:**
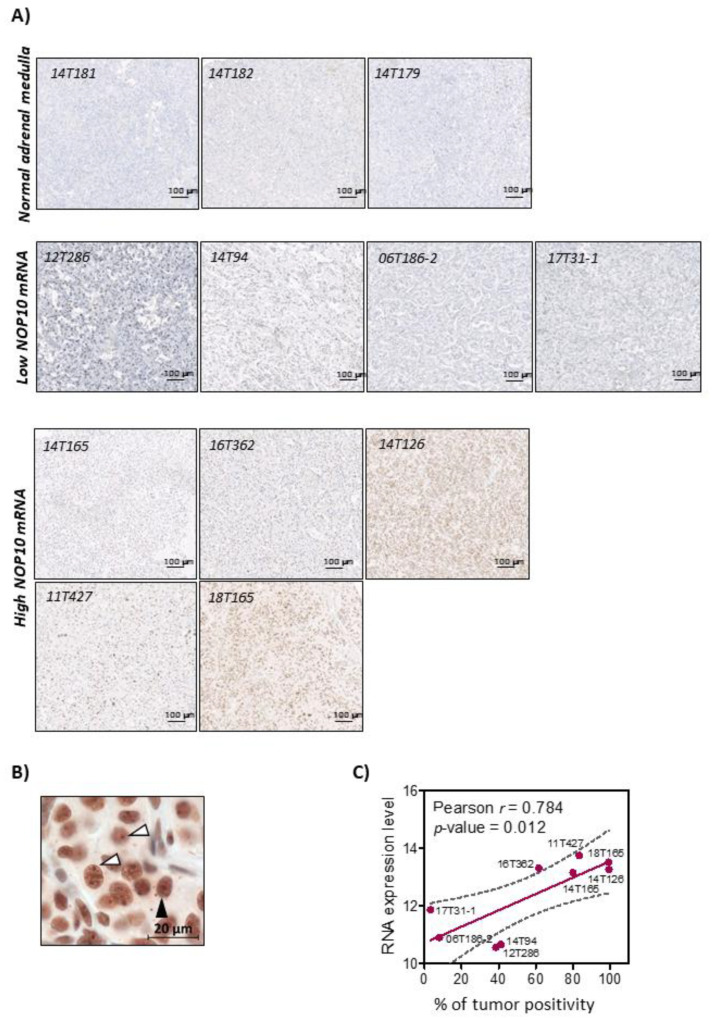
NOP10 immunohistochemistry. (**A**) Representative staining images of normal adrenal medulla (*n* = 3), tumors with low *NOP10* expression (*n* = 4) and *NOP10* overexpressing tumors (*n* = 5). (**B**) Magnified image from an NOP10-positive staining (14T126). Black arrow: representative nuclear staining. White arrows: representative nucleolar staining. (**C**) Linear regression plot of *NOP10* RNA expression and percentage of tumor positivity. Pearson correlation r and *p*-value are shown.

Regarding *NOP10*, no pathogenic mutations were found that could explain its upregulation, neither in the exons nor in the studied part of the gene promoter (200 bp upstream the TSS). Furthermore, no significant correlation was found between *NOP10* expression and the methylation status of any of the nine CpG sites studied at the gene locus (2 CpG) or promoter region (7 CpG) (up to 230 bp from TSS), according to TCGA and CNIO data sets. Similarly, the expression of miRNAs with conserved binding sites at the *NOP10* locus, according to TargetScan tool (http://www.targetscan.org/vert_72/, accessed on 17 February 2021), miRNAs -204, -211, -194, -27, -128 and -135 did not inversely correlate with *NOP10* expression. Additionally, no alterations in the number of copies for the *NOP10* locus were detected in our sample set. Therefore, none of the canonical mechanisms associated with an altered gene expression underlay the significantly higher *NOP10* expression levels found in mPPGLs (Figure 2). Nevertheless, a selection of samples with high *NOP10* expression showed a significantly higher NOP10 staining at the nuclear and nucleolar levels by immunohistochemistry (Figure 3A,B). Moreover, NOP10 IHC staining and gene expression were highly correlated (Pearson r = 0.784; *p*-value: 0.012) (Figure 3C).

### 3.4. Telomerome Significant Genes Identification and Predictive Value

The risk given by the two candidate genes (*NOP10* and *FBXO4*) was evaluated using a univariate logistic regression model, as well as *TERT*+*ATRX* as a single variable (bona fide markers of immortalization) and *SDHB* as a genetic variable associated with worse prognosis. The univariate regression model revealed that each of them were associated with a higher risk to develop metastatic disease. Finally, a step-wise model selected *TERT*+*ATRX* and *NOP10* as the best classifier of metastasis (Table S3). *FBXO4* and *SDHB* were excluded from the model, suggesting that they did not confer malignancy by themselves.

**Figure 4:**
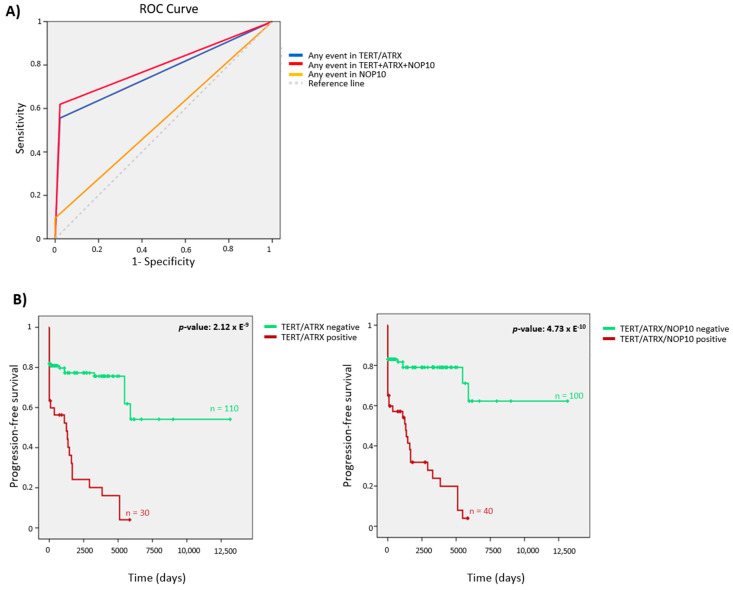
(**A**) Receiver operating characteristic curve (ROC) analysis showing the accuracy of telomerome events to distinguish between metastatic and non-metastatic samples. This data corresponds to all metastatic (*n* = 54) and non-metastatic cases with ≥8 years of follow-up (*n* = 45). Metastases (*n* = 9), clinically aggressive samples (*n* = 5) and non-metastatic cases with <8 years’ follow-up (*n* = 52) were excluded. Genes were introduced as a dichotomous variable based on outlier expressors. *TERT* events: overexpression, promoter mutation, promoter hypermethylation or gains; *ATRX* events: low expression outliers and mutations; *NOP10* events: overexpression outliers. Any event in *TERT*+*ATRX*: *p*-value: 2.46 × E^−6^, AUC: 0.767; 95%CI: 0.678–0.856; any event in *TERT*+*ATRX*+*NOP10*: *p*-value: 1.35 × E^−7^, AUC: 0.798; 95%CI: 0.714–0.882; any event in *NOP10*: *p*-value: 0.439, AUC: 0.548; 95%CI: 0.439–0.656. (**B**) Kaplan–Meier plots of time to progression of patients, according to the events in *TERT*/*ATRX* (left) and to the events in the three telomerome significant genes (*TERT*/*ATRX*/*NOP10*) (right). *n* = number of samples. Log-rank test *p*-value is shown. Non-metastatic patients with unknown follow-up and those with clinically aggressive tumors were excluded from the analysis.

We applied the AUC analysis to determine the metastatic risk predictive value of the selected genes. Although events in *TERT*/*ATRX* explained a significant number of metastatic cases (AUC = 0.767, 95%CI = 0.678–0.856, *p*-value = 2.46 × E^−6^), the *TERT*/*ATRX*/*NOP10* combination was a better predictor (AUC = 0.798, 95%CI = 0.714–0.882, *p*-value = 1.35 × E^−7^), suggesting that *NOP10* aberrant expression contributes to the PPGL progression (Figure 4A). In addition, patients carrying alterations in *TERT*/*ATRX*/*NOP10* showed a significant shorter time to progression than those without events (*p*-value: 4.73 × E^−10^, HR: 5.05, 95%CI: 2.76–9.23) (Figure 4B).

### 3.5. TERT, ATRX and NOP10 Events Affect Telomere Length

We measured telomere length in samples with *TERT*, *ATRX* and *NOP10*-altered profiles by Q-FISH technique. Additionally, three non-metastatic samples without any alterations in any of these genes and three normal adrenal medullae were included as controls.

**Figure 5:**
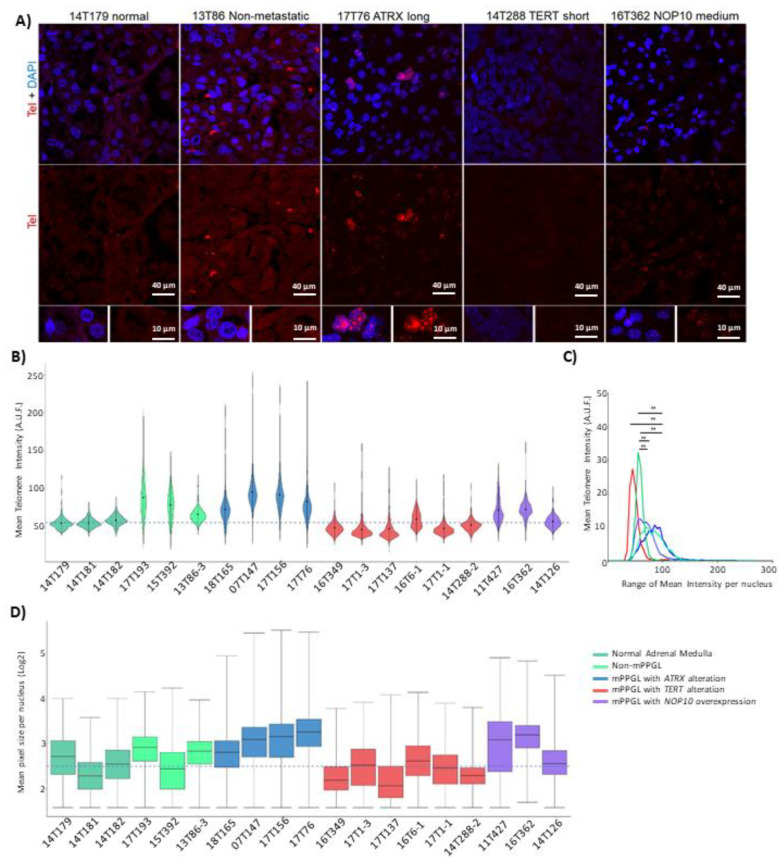
(**A**) Representative Q-FISH images from different tumors. 14T179: normal adrenal medulla with short telomeres. 13T86: non-metastatic PPGL with short telomeres. 17T76: mPPGL with *ATRX* mutation (c.3622dup, p.Ile1208AsnfsTer4) and long telomeres. 14T288: mPPGL with extremely short telomeres, this patient has *TERT* overexpression, promoter hypermethylation and 5p amplification. 16T362: mPPGL with medium-long telomeres and *NOP10* overexpression. (**B**) Violin plot of telomere mean intensity per nucleus. Highest values (upper end) represent long telomeric regions. Black dots inside each violin box represent median intensity value. Dashed line represents the median value of normal samples intensity (normal adrenal medulla, *n* = 3). Non-mPPGL: 17T193 (*FGFR1*-mutated); 15T392 (WT); 13T86-3 (*SDHB*-mutated). (**C**) Mean telomere intensity distribution for each group of samples (Wilcoxon matched-pairs signed rank test, Gaussian Approximation). (**D**) Box plot representing the mean telomere size (mean pixel size per nucleus) for each tumor: *ATRX* mutants have extremely long telomeres, *NOP10*-altered samples show intermediate-long telomeres, *TERT*-altered present extremely short telomeres. Normal and non-metastatic samples have medium-short telomeres. Dashed line represents the median value of normal samples’ telomere size. The color code chart applies to panels **B**, **C** and **D** (one-way ANOVA Tukey’s multiple comparison test: **: *p*-value < 0.01).

Confocal microscopy analysis revealed that tumors harboring *TERT* alterations had shorter telomeres than the controls. Those with *ATRX* mutations presented higher heterogeneity in the telomere length, as observed by the wider telomere distribution shown and the high number of extremely long telomeres. Tumors overexpressing *NOP10* showed an intermediate phenotype between short and long telomeres (Figure 5A,B). Differences in the distribution of telomere average intensity between groups were statistically significant, showing a higher frequency of long telomeres in *ATRX* mutants and *NOP10*-altered samples compared with the normal and non-metastatic ones, and a higher frequency of short telomeres in *TERT*-altered samples (Figure 5C). Differences in the mean of telomere spot size were also observed, confirming the results obtained with the mean telomere intensity analysis (Figure 5D).

The alternative telomere lengthening (ALT) phenotype is characterized by the high heterogeneity of telomere length and the presence of extremely long telomeres. ALT has unequivocally been associated with *ATRX* alterations [52,53]. We analyzed the colocalization of PML nuclear bodies with telomeres (ALT-associated PML nuclear bodies or APBs), a phenomenon previously described in ALT-positive cells with increased telomere recombination [54]. Representative samples were selected for APB assays (Figure S3A). The percentage of PML-positive cells was significantly higher in *ATRX* mutated samples as compared to *ATRX* WT ones (Figure S3B). In addition, a larger number of APBs was observed in the *ATRX* mutants (Figure S3C). Samples without *ATRX* mutations did not show APBs, whereas three out of four *ATRX* mutant samples presented a high number of APBs and were therefore classified as ALT^+^. Interestingly, samples with *NOP10* alteration presenting intermediate/long telomeres, though showing PML-positive cells (5%) did not present APBs (Figure S3A–C), ruling out ALT mechanism in *NOP10* overexpressing samples.

### 3.6. NOP10 and TERT Expression in Primary Cultures Affect Telomere Length Maintenance

To determine the role of *NOP10* overexpression in cell immortalization and telomere lengthening, primary cultures from human umbilical cord mesenchymal stem cells (hUCMSC) were modeled to overexpress either *NOP10*, *TERT* or both genes simultaneously (Figure S4A,B). An unmodified primary culture (parental) of hUCMSC was used as the control condition.

**Figure 6:**
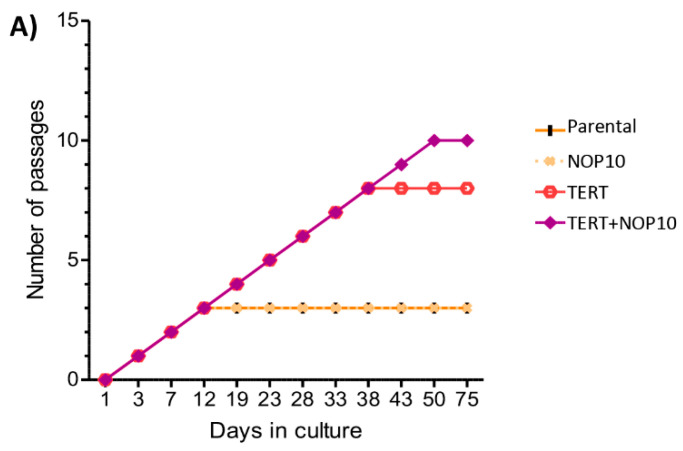

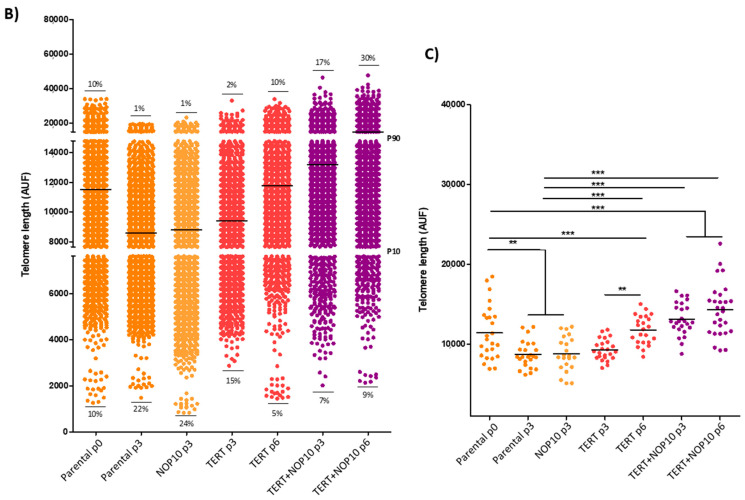
*In vitro* telomere length analysis. (**A**) Cell proliferation per condition. X axis: number of days in culture since antibiotic selection; Y axis: accumulative number of passages. Parental and *NOP10* become quiescent after 3 passages. *TERT* cells become quiescent after 8 passages and *TERT*+*NOP10* after 10 passages. (**B**) Scatter dot plot showing telomere length. Percentages of short and long telomeres for each isogenic primary culture are shown. Graph separations were made according to percentile 10 and 90 (P10 and P90) based on “Parental p0”. Median telomere length value graphed in black. (**C**) Median telomere length value per cell (one-way ANOVA Tukey’s multiple comparison test: **: *p*-value < 0.01; ***: *p*-value < 0.001).

Cell growth curve analysis of the isogenic primary cultures showed that both the parental control and *NOP10* cells became quiescent/non-replicative after three passages, acquiring an expanded/quiescent morphology. *TERT* expression alone or in combination with *NOP10* delayed the non-replicative status until passage 8 and 10, respectively, in accordance with a higher number of fibroblastic/dividing cell morphology in both conditions (Figure 6A and Figure S4A–C).

Analysis of telomere length by Q-FISH of all isogenic primary cultures at passage three, when parental and *NOP10* conditions reached the replicative quiescent state (Figure 6A), showed that both cultures presented equally short telomeres and a similar percentage of critically short telomeric signals (>20% below 10th percentile of parental cells) (Figure 6B, Figure S4A,C). In contrast, *TERT* overexpressing cells presented a progressive reduction in the percentage of short telomeres and an increase in median telomere length from passage three to passage six (*p*-value < 0.001), indicating a *TERT*-dependent telomere lengthening (Figure 6A,B). Notably, *TERT+NOP10* overexpressing primary cultures had the longest median telomeric lengths of all the tested conditions (*p*-values < 0.001), suggesting an enhanced effect of *TERT* and *NOP10* on telomere length maintenance (Figure 6B,C). Indeed, the proportion of long telomeres (>90th percentile) was 3-fold higher in *TERT*+*NOP10* cells as compared to *TERT* cells at passage six (Figure 6B,C).

## 4. Discussion

To date, some PPGL-specific markers have been described [8,10,11,12,41,42,43,45]. However, the problem still facing PPGL patients is the lack of molecular markers capable of predicting the development of metastases at an earlier stage. PPGL patients can develop metastases up to 10 years after the diagnosis of the first tumor, and any PPGL should be considered as potentially metastatic, as the most recent WHO classification states [34,55].

Additionally, mechanisms that appear widely de-regulated in cancer, such as cell immortality, have also been described in PPGL [56]. In this regard, there is sufficient evidence to support the association of *TERT* and *ATRX* alterations with disease progression [14]. In this work, we aimed to analyze the prognostic value of these and other additional genes involved in this biological process.

Our results are in consonance with previous studies: *TERT* expression is commonly mediated by genetic and epigenetic mechanisms [3,14,15,57,58] and only detectable in metastatic PPGL but not in non-metastatic cases [59]. In addition, among all the mechanisms associated with *TERT* expression, we found that copy number gains of *TERT* locus showed the highest levels of *TERT* transcriptional activation [14,15,60,61]. Similarly, rearrangements in the *TERT* promoter have been reported to be associated with high levels of *TERT* expression [60]. This mechanism could explain the data observed in three *TERT*-WT samples of our series, which showed equally high levels as samples with *TERT* locus gains. Additionally, some samples that harbor events involving *TERT* have not shown significant changes in *TERT* expression levels. However, the prognostic value of these alterations remains significant, as they are almost exclusive of mPPGLs. We found that mutations in *ATRX* were also exclusive of mPPGLs and associated with a decreased expression [14,62,63].

We validated the distribution of *TERT* and *ATRX* events among PPGL genetic classes [14,64,65,66]. Most of these samples belong to C1A, although they are not exclusive of this cluster nor *SDHB*-mutant tumors, as previously reported [14,15]. These data reinforce previous evidence of the role of *TERT* and *ATRX* as prognostic markers in PPGL [13,67,68,69,70].

Additionally, our study identified *NOP10* overexpression as a novel prognostic marker in PPGL. Probably both the size of the series of available metastatic cases, the extensive follow-up time for a considerable number of patients and the comprehensive analysis of genes related to telomere maintenance have allowed the identification of *NOP10* as a new risk marker, which until now had gone unnoticed in other previous studies. In this regard, a pan-cancer study based on the systematic analysis of immortalization mechanisms identified the TCGA-PPGL series as a tumor type with limited occurrence of immortalization hallmarks [56]. This is probably due to the reduced number of metastatic cases of this latter series in comparison to ours. Our prognostic model prioritized alterations in telomere maintenance genes (*TERT*+*ATRX*+*NOP10*) over *SDHB*-mutation status. The *SDHB* prognostic role has already been questioned when a comprehensive set of clinico-pathological features was considered [13]. Notably, the *TERT*+*ATRX*+*NOP10* combination identified the group of patients with the shortest time to progression in our series.

*NOP10* belongs to the family of H/ACA small nucleolar ribonucleoproteins (snoRNPs), which is also comprised by DKC1, NHP2 and GAR1. These snoRNPs have a constitutive expression at the nuclear and nucleolar level and have two functions: as part of the telomerase complex, they are involved in its stabilization [71,72], and they also participate in rRNA post-transcriptional modifications through pseudourydilation [73,74].

Highly positive NOP10 IHC staining has been already associated with shorter time to progression and aggressiveness in lung and breast cancer [75,76,77,78]. Our IHC results have demonstrated that *NOP10* expression outliers have a highly positive staining. The good correlation between expression and IHC supports the implementation of NOP10 immunohistochemistry as an additional prognostic tool. Additionally, NOP10 protein is located both at the nuclear and nucleolar level, suggesting the dual role of NOP10 in our mPGGL.

Regarding the role of *NOP10* in telomere maintenance, our *in vitro* results showed that *NOP10* on its own has not had a direct effect on the telomere length. However, when *NOP10* overexpression coexists with *TERT*, telomeres lengthen and cells delay the entrance on a quiescent state. In agreement with this finding, Q-FISH analysis of telomeric lengths on tumors showed that the mPPGL 16T362, with a *TERT* copy gain and *NOP10* overexpression, had a significantly higher median telomeric length as compared to that of mPPGLs bearing only *TERT* alterations. In addition, *NOP10* overexpressing mPPGLs displayed a higher mean telomere length and a lower percentage of short telomeres compared with *TERT*-only mPPGLs. None of these samples were classified as ALT(+), strongly supporting a *NOP10* role in facilitating telomerase-dependent telomere lengthening in these tumors.

Given the fact that *NOP10* is involved in the anchorage of the telomerase complex to the Cajal bodies [79,80], we speculate that the overexpression of this protein could help to generate a more durable interaction favoring telomere lengthening. However, based on our *NOP10* nucleolar staining, we cannot rule out additional indirect effects in telomere lengthening through RNA stabilization.

One of the limitations of this study, as occurs in many other tumors, is that we cannot rule out that intratumoral heterogeneity is limiting the discriminatory ability of our analysis. Therefore, it is plausible that we are not detecting the immortalization markers reported in this study in some of the PPGLs.

## 5. Conclusions

In summary, we showed that *NOP10* is a novel metastatic risk marker in PPGLs, which in combination with alterations in *TERT* and *ATRX*, provided the strongest means of stratification in our series, independently of *SDHB*-mutation status. In *NOP10* overexpressing tumors, we observed an intermediate-length telomere phenotype without ALT, which together with *in vitro* results, suggest that *NOP10* has a role in telomerase-dependent telomere maintenance.

We propose to include NOP10 immunostaining within the current battery of markers for stratifying PPGL patients to fine-tune their prognosis, thereby providing early detection of metastatic disease and ultimately bettering the planning of treatment options.

## Figures and Tables

**Table 1:** Summary of PPGL series clinical data.

Characteristics	Patients
**CNIO series (*n* = 149)**	
Gender	
Female	54.4% (81)
Male	43% (64)
Unknown	2.7% (4)
Age at initial diagnosis of PCC/PGL; (range) in years	
	45 (9–82)
Cluster	
C1A	40.3% (55)
C1B	9.4% (14)
C2	36.9% (54)
C3	4.7% (7)
WT	13.4% (19)
Clinical behavior	
Metastatic	34.6% (47)
Synchronous	19.9% (28)
Metachronous	14.7% (19)
Clinically aggressive	3.2% (5)
Non-metastatic	62.2% (97)
Driver	
*SDHB*	21.2% (30)
Tumor type	
PCC	54.4% (81)
PGL	29.5% (44)
Bilateral PCC	3.4% (5)
Multiple PGL	3.4% (5)
PCC+PGL	7.4% (11)
Unknown	2% (3)

## Data Availability

Data will be available upon reasonable request.

## Supplementary Materials

The following are available online at https://www.mdpi.com/article/10.3390/cancers13194758/s1, Figure S1: Print of telomerome expression outliers, Figure S2: Mechanisms altering TERT expression, Figure S3: APBs detection, Figure S4: Summary of in-vitro experiment. Table S1: Summary of the mutations found in *ATRX* by NGS (exome sequencing and customized panel). Table S2: Summary of the primers used for NGS in blue, Sanger sequencing in green and RT-PCR in purple, Table S3: Univariate logistic regression analysis and stepwise conditional logistic regression model to assess the odds of metastasis risk.
