# Supplementary material for: Analysis of Telomere Maintenance Related Genes Reveals NOP10 as a New Metastatic-Risk Marker in Pheochromocytoma/Paraganglioma
Source: Cancers (Basel). 2021 Sep 23;13(19):4758. doi: 10.3390/cancers13194758 (PMC8507560; doi:10.3390/cancers13194758)
Supplement: Supplementary file 1 [file cancers-13-04758-s001.zip › cancers-1375820-Suplementarias 21.09.2021.pdf]

Sample	ATRX mutation	Platform	Expression Level
17T76	c.3622dup, p.Ile1208AsnfsTer4	WES	8.84*
12T232	c.6252C>A ;p.Tyr2084Ter	NGS customized panel	9.27*
17T156	c.5268_5272del	WES	9.91*
18T165	c.1493_1496del; p.Arg498LysfsTer15	WES	10.18
17T77-1	c.1094del;p.Asn365ThrfsTer2	WES	10.28
07T147	c.1441G>T; p.Glu481Ter	WES	10.82

Supplementary Table S1. Summary of the mutations found in *ATRX* by NGS (exome sequencing and customized panel). Expression outliers are marked with an asterisk (*).

Primer name	Type	Sequence
**TERT promoter (NGS)**	Forward	TCGTCGGCAGCGTCAGATGTGTATAAGAGACAG**CAGCGCTGCCTGAAACTCG**
	Reverse	GTCTCGTGGGCTCGGAGATGTGTATAAGAGACAG**GTCCTGCCCCTTCACCTTC**
**THOR_A1 (NGS)**	Forward	TCGTCGGCAGCGTCAGATGTGTATAAGAGACAG**GGAGGGGTTGGGAGGGTT**
	Reverse	GTCTCGTGGGCTCGGAGATGTGTATAAGAGACAG**CCTACCCCTTCACCTT**
**THOR_A2 (NGS)**	Forward	TCGTCGGCAGCGTCAGATGTGTATAAGAGACAG**AGTTGGAAGGTGAAGGGGTAGG**
	Reverse	GTCTCGTGGGCTCGGAGATGTGTATAAGAGACAG**AACTCCCAATAAATTC**
**THOR_A3 (NGS)**	Forward	TCGTCGGCAGCGTCAGATGTGTATAAGAGACAG**GAATTTATTGGGAGTT**
	Reverse	GTCTCGTGGGCTCGGAGATGTGTATAAGAGACAG**TCCCTACACCCTAAAAA**
**THOR_A4 (NGS)**	Forward	TCGTCGGCAGCGTCAGATGTGTATAAGAGACAG**GTTTAGGTTGTGGGGTAATT**
	Reverse	GTCTCGTGGGCTCGGAGATGTGTATAAGAGACAG**CTAAAAACAACCCTAAATC**
**TERT promoter (Sanger)**	Forward	GTCCTGCCCCTTCACCTTC
	Reverse	CAGCGCTGCCTGAAACTC
**NOP10 exon 1 (Sanger)**	Forward	GCAGGAAGGAAATTGACGAA
	Reverse	CGTATGACCTCACCCACTCC
**NOP10 exon 2 (Sanger)**	Forward	AGCAGAAATTTGACCCGATG
	Reverse	TAAGGGACCCTCAGAGGACA
**TERT (RT-PCR) UPL #18**	Forward	CGGTGTGCACCAACATCTAC
	Reverse	GCACACATGCGTGAAACCT
**NOP10 (RT-PCR) UPL #15**	Forward	GGGACAACAGACCTGCTCA
	Reverse	GGTGATTCGGTGTCGAGAGT
**β-ACTIN (RT-PCR) UPL #11**	Forward	ATTGGCAATGAGCGGTTC
	Reverse	CGTGGATGCCACAGGACT

Supplementary Table S2. Summary of the primers used for NGS in blue, Sanger sequencing in green and RT-PCR in purple. NGS primers specific sequence in bold, common adapters in regular type.

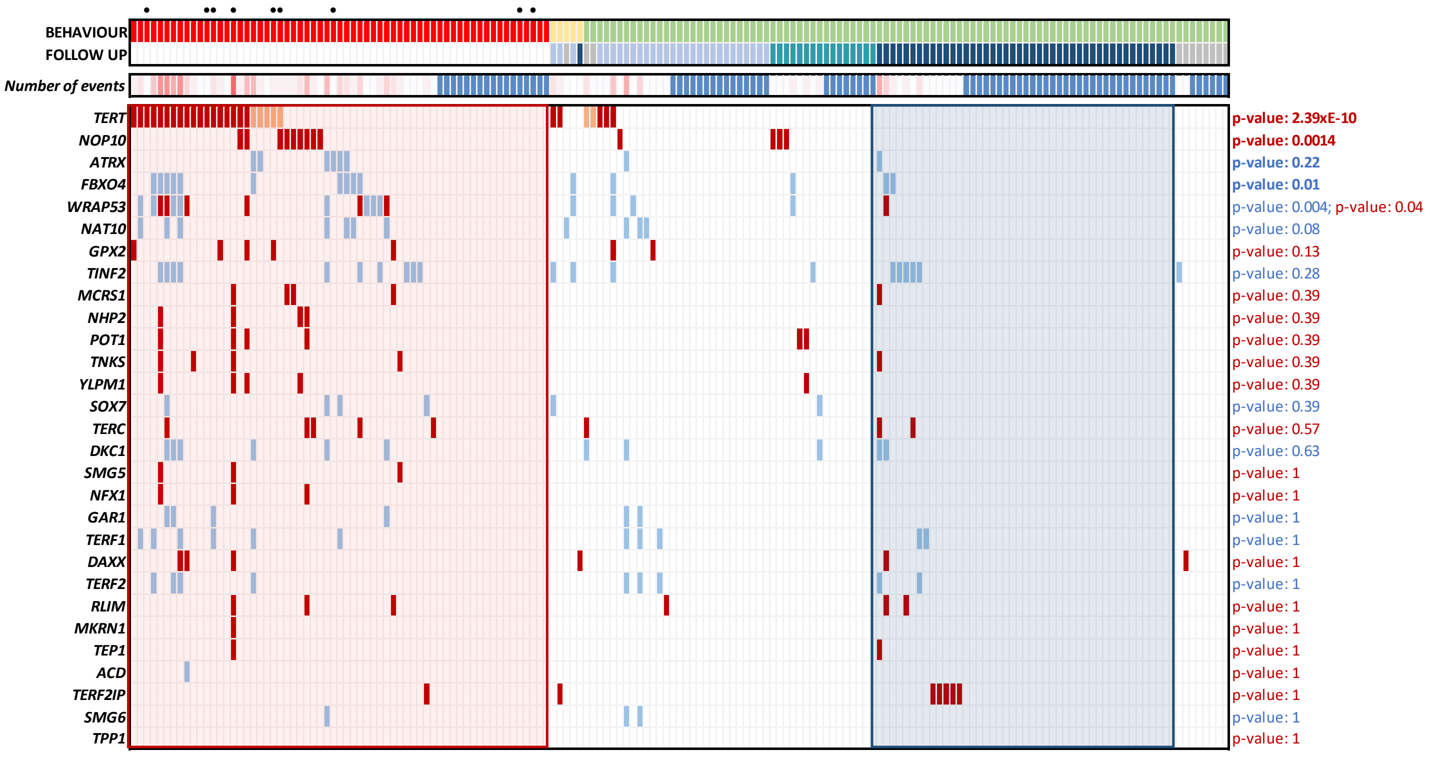

Supplementary Figure S1. Print of *telomerome* expression outliers. Bonferroni corrected p-values (Fisher exact test; in red: over Q3+(1,5 x IQR) and in blue: below Q1-(1,5 x IQR)). Thresholds were established comparing mPPGL (red rectangle) with >8 years of follow-up tumors (blue rectangle). mPPGL (N=63); non-mPPGL >8 years of follow-up (N=45). Black dots indicate metastasis.

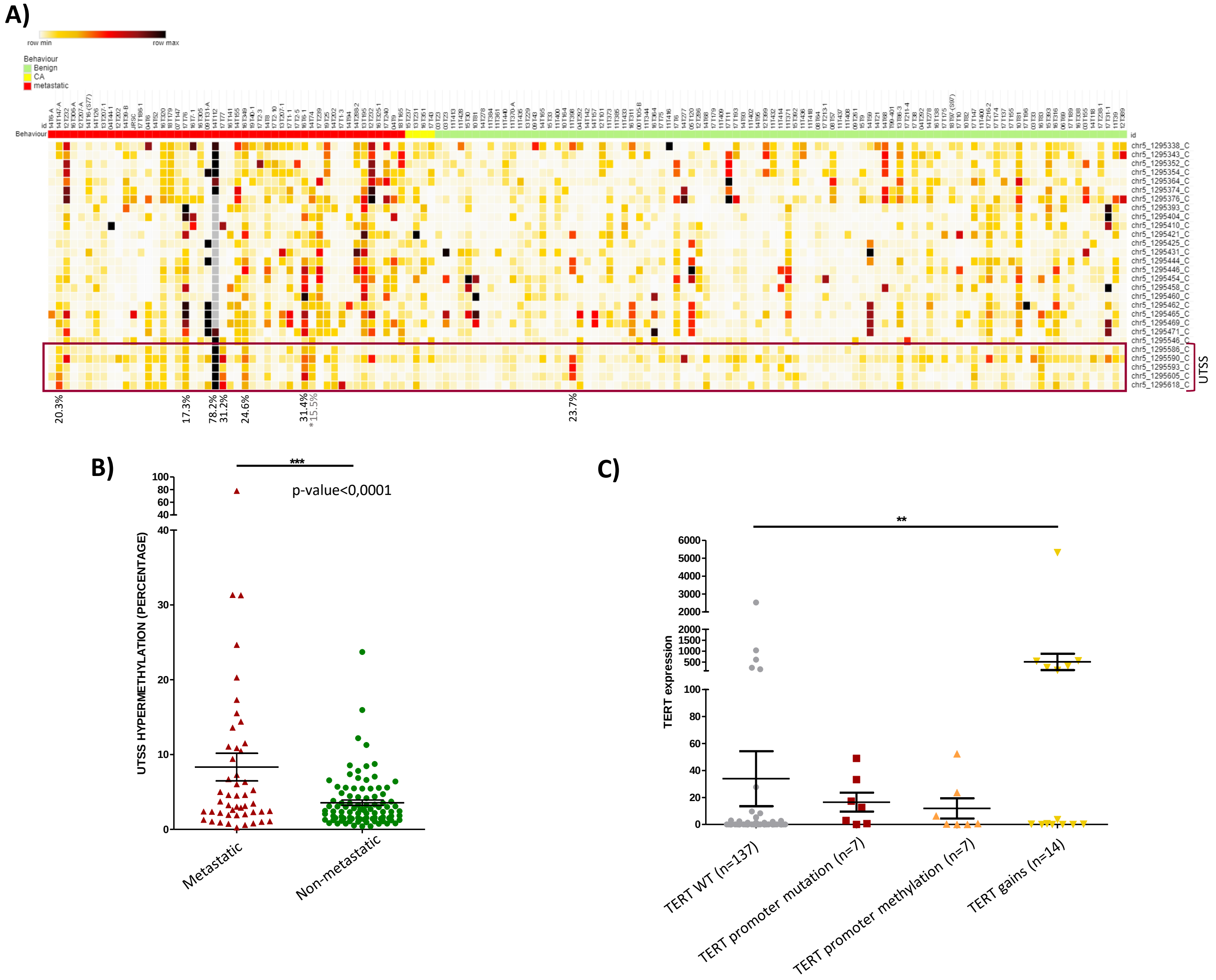

Supplementary Figure S2. Mechanisms altering *TERT* expression: **A)** Heatmap of *TERT* promoter methylation levels in PPGL. UTSS methylation average value is shown for those samples beyond threshold (≥16,1%). **B)** Percentage of UTSS methylation in metastatic (n=48) and non-metastatic samples (n=93) (clinically aggressive were excluded from the analysis). **C)** Correlation of *TERT* expression values with *TERT* different events. 5p gains are significantly associated with higher expression levels.

Univariate logistic regression				
Variable	OR	I.C. 95%		p-value
		Lower	Upper	
SDHB	4.48	1.773	11.319	0.002
TERT+ATRX	25.625	8.211	79.973	2,33x E^{-8}
NOP10	3.565	1.018	12.486	0.047
FBXO4	4.65	1.318	15.718	0.013

Stepwise logistic regression				
Variables	OR	I.C. 95%		p-value
		Lower	Upper	
TERT+ATRX	28.5	8.963	90.623	1.38x E^{-8}
NOP10	5.346	1.34	21.32	0.0175

Supplementary Table S3. Univariate logistic regression analysis and stepwise conditional logistic regression model to assess the odds of metastasis risk. Each variable was dichotomized according to the presence or absence of any of the analyzed alterations.

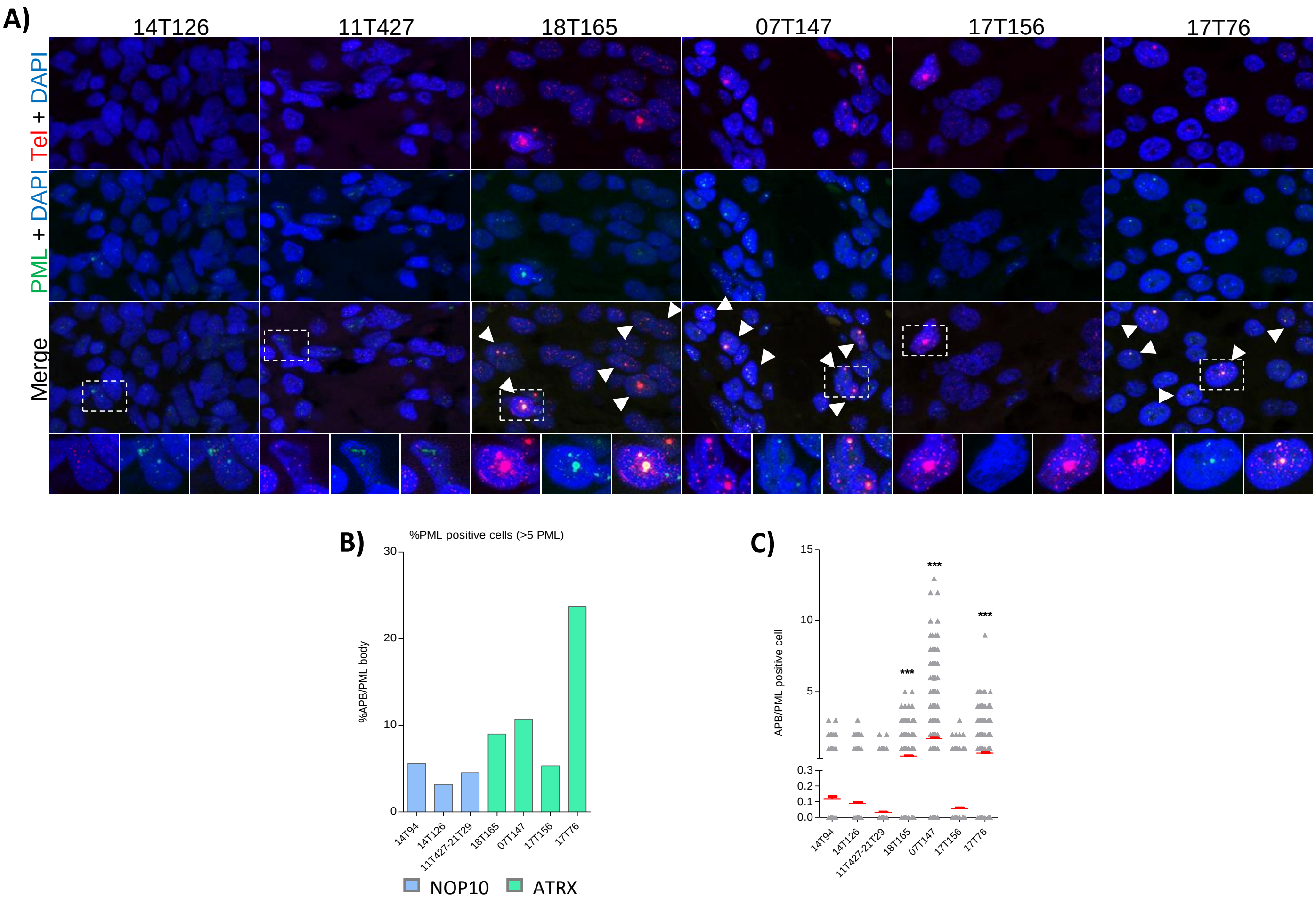

Supplementary Figure S3. APBs detection. **A)** Representative Immuno-FISH images of long telomere samples detected by Q-FISH. PML bodies are present in every sample at different proportions (<5 PML are not considered). *NOP10* altered samples show long telomeres but no colocalization with PML bodies was detected. *ATRX* mutant PPGL show extreme long telomeres which colocalize with PML bodies (white arrows pointing APBs). 17T156 sample present the same long telomeres but any APB was detected. **B)** Percentage of PML bodies per cell. Higher number of PMLs are detected in *ATRX* mutants. **C)** Number of APBs (colocalizations) detected per cell in each sample. Red line represents the median value.

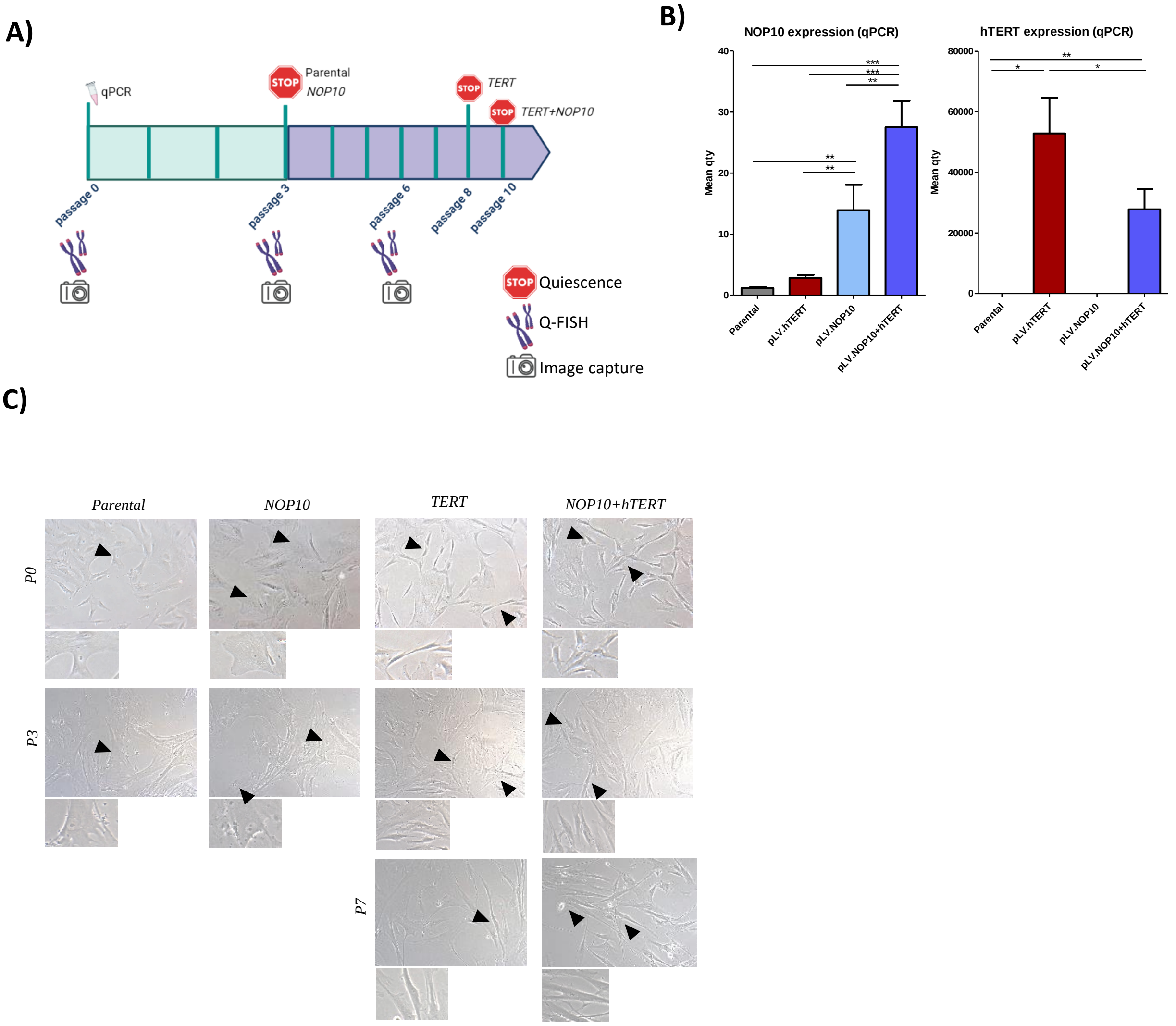

Supplementary Figure S4. Summary of in-vitro experiment. **A)** Overall view of the protocol followed in the cell culture experiment. Passage 0: RT-qPCR and Q-FISH for all the conditions. Passage 3: Q-FISH for all the conditions; Parental and *NOP10* become quiescent, *TERT* and *TERT+NOP10* continue dividing. Passage 6: Q-FISH for *TERT* and *TERT+NOP10*. Passage 8: *TERT* become quiescent. Passage 10: *TERT+NOP10* become quiescent **B)** RT-PCR of *NOP10* and *hTERT* expression levels in cells after infection for each experimental condition (One-way ANOVA). **C)** Representative images of cell proliferation in each passage. Black arrows showing quiescent cells: expanded morphology (Parental and *NOP10)*; dividing cells: fibroblastic morphology and spherical refringent spots (*TERT* and *TERT+NOP10*).
